# The prognostic significance of the geriatric nutritional risk index in postoperative parotid gland carcinoma

**DOI:** 10.1038/s41598-025-08609-8

**Published:** 2025-07-01

**Authors:** Xue-Lian Xu, Hao Cheng

**Affiliations:** 1 Department of Radiotherapy Oncology, The First Affiliated Hospital of Xinxiang Medical University, Xinxiang, 453100 Henan China; 2 Department of Radiotherapy Oncology, Affiliated Cancer Hospital of Zhengzhou University, Zhengzhou, 450000 Henan China

**Keywords:** Parotid gland carcinoma, Geriatric nutritional risk index, Nomogram, Postoperative, Prognosis, Cancer models, Head and neck cancer, Cancer models, Head and neck cancer, Cancer, Surgical oncology, Cancer, Cancer

## Abstract

The impact of the Geriatric Nutritional Risk Index (GNRI) on the prognosis of postoperative parotid gland carcinoma (PGC) remains unclear. This study investigates the role of GNRI in predicting disease-free survival (DFS) and overall survival (OS) in postoperative PGC patients and develops a predictive model. We conducted a retrospective analysis of 389 postoperative PGC patients treated from May 2008 to June 2019 at two regional medical centers in China. The independent prognostic factors were identified by univariate and multivariate Cox regression analyses. Then, two nomograms were established based on these independent prognostic factors and verified by a series of methods. The GNRI was identified as an independent prognostic factor in postoperative patients with PGC. A higher GNRI value indicated a worse prognosis for the patient. Based on the recognized independent prognostic factors—such as surgical margin, perineural invasion, extranodal extension (ENE), age-adjusted Charlson comorbidity index (ACCI), American Joint Committee on Cancer (AJCC) stage, and GNRI—two nomograms for overall survival (OS) and disease-free survival (DFS) were developed. The concordance index (C-index) for DFS was 0.712 and 0.730, while for OS it was 0.697 and 0.722 in the training and validation groups, respectively. These results demonstrate a significant improvement over the traditional AJCC staging system. Additionally, the area under the curve (AUC) values in both the training and validation sets were impressive, with all AUC scores exceeding 0.7. Decision curve analysis (DCA) also indicated substantial clinical benefits. The GNRI is an important predictive index in postoperative patients with PGC, which is not limited to the elderly. The newly developed nomograms provide a new tool of prognostic evaluations for postoperative patients with PGC.

**Supplementary Information:**

The online version contains supplementary material available at 10.1038/s41598-025-08609-8.

## Inrtoduction

As the largest salivary gland in the human body, the parotid gland is involved in the moist and initial digestion of the mouth and the protection of oral health^1^. Parotid gland carcinoma (PGC) is a rare tumor and the most common type of major salivary gland carcinoma^2^, with diverse histological subtypes and a relatively low incidence^3^. Epidemiological studies indicate that parotid gland cancer occurs more frequently in individuals over the age of 50^4^. Radical surgical resection is the primary treatment for PGC patients^5^. Since parotid gland plays an important role in human nutrition intake, postoperative patients with PGC should pay more attention to the evaluation of nutritional status. Although the role of nutrition-related indicators in the prognosis of postoperative patients has gained attention among clinicians, these indicators have not yet been included as quantitative measures in prognostic evaluation systems. The prognostic factor for postoperative PGC patients is still being explored and controversial.

Currently, the American Joint Committee on Cancer (AJCC) staging system is the most widely used method for prognostic assessment in PGC patients, updated in its latest 8th edition^6,7^. As we learn more about PGC, we find additional factors that can affect the prognosis of patients with PGC, which are not included in the traditional staging system. The traditional AJCC staging system complicates the assessment of how factors such as age, comorbidities, tumor location, pathological type, grade, surgical margins, nutritional status, and others influence the prognosis of PGC^8^. Individualized assessment of the survival rate for PGC patients is challenging. Therefore, a more effective tool is needed for predicting prognosis of PGC patients to address this issue. Nomograms are widely used tool in oncology^9–13^, allowing for the integration of various variables to estimate the survival rate. This tool meet the need for a comprehensive clinical model and support personalized medicine approaches^8,14,15^.

The Geriatric Nutritional Risk Index (GNRI) is a straightforward nutritional screening tool that assesses the nutritional status of elderly patients based on serum albumin concentration and body weight. Introduced by Bouillanne et al. in 2005, the GNRI was initially developed to evaluate the nutritional status of elderly patients in hospitals and to predict risks associated with malnutrition^16^. Its simple calculation method makes it easy to use in clinical practice. Research has shown that the GNRI is effective in predicting hospitalization duration, complications, and mortality rates among older patients. Over time, its application has expanded to include a wider range of malignant tumors and various age groups^17–21^. Nevertheless, the effect of GNRI on the prognosis of patients following PGC surgery remains unclear.

This study aims to evaluate the impact of GNRI on the prognosis of postoperative patients with PGC. Then, developing new predictive nomogram models integrating GNRI for postoperative patients with PGC to improve the efficiency of individualized prognosis assessment.

## Materials and methods

### Materials

**Fig. 1:**
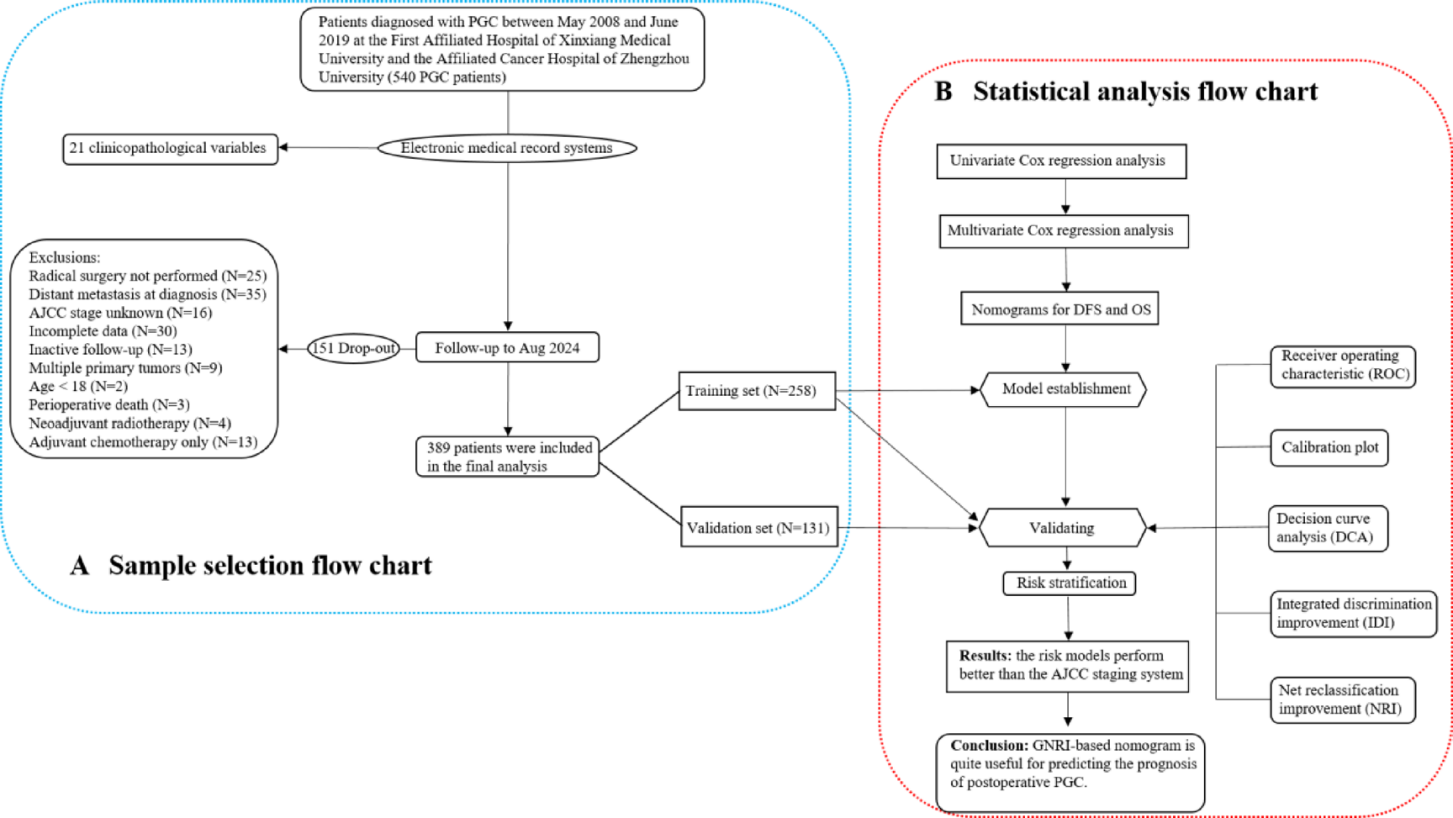
Study flow chart. Diagram **A** outlines the sample selection process, while Diagram **B** presents the statistical methodology flow. AJCC, American Joint Committee on Cancer; DFS, disease-free survival; OS, overall survival; PGC, parotid gland carcinoma.

This retrospective study included 389 patients treated between May 2008 and June 2019 at two regional medical centers in central China. The inclusion criteria were as follows: age ≥ 18 years; confirmation of pathological diagnosis; and the presence of primary malignant tumors in the parotid gland. Patients were excluded based on the following criteria: radical surgery not performed, distant metastasis at diagnosis, unknown AJCC stage, incomplete data, inactive follow-up, multiple primary tumors, age under 18, perioperative death, neoadjuvant radiotherapy, and adjuvant chemotherapy. The sample screening flow chart is shown in Fig. 1A. All participants gave informed consent before joining the study, and the research protocol received approval from the institutional ethics committee. The radiotherapy methods used in treatment included conformal radiotherapy (CRT), intensity-modulated radiotherapy (IMRT), and volumetric-modulated arc therapy (VMAT). The prescribed total radiation dose ranged from 60.0 to 70.0 Gy, delivered in fractionated doses of 2.0 to 2.2 Gy per session. Treatment sessions were conducted once daily, five days a week, following standard protocols. Tumor staging was performed according to the 8th edition of the AJCC staging system, utilizing postoperative pathological staging when applicable. For patients treated prior to 2016, staging was retrospectively restaged according to the 8th edition guidelines to maintain consistency across the study period.

### Variables collection

The study analyzed a total of 21 clinicopathological variables. These variables included: age at diagnosis, gender, pathology, tumor grade, tumor location, smoking habits, Eastern Cooperative Oncology Group (ECOG) performance status (PS) score, surgical margin status, evidence of perineural invasion, vascular invasion (VI), extranodal extension (ENE), 8th edition AJCC stage, body mass index (BMI), hemoglobin levels, albumin levels, Geriatric Nutritional Risk Index (GNRI), age-adjusted Charlson comorbidity index (ACCI), type of resection, adjuvant radiotherapy, disease-free survival (DFS), and overall survival (OS). The primary endpoints of the study were DFS and OS.

### Calculation formula

The formulas for BMI, GNRI, and ACCI used in this study are as follows:

$${\text{BMI}}\,=\,{\text{Weight }}\left( {{\text{kg}}} \right)/{\text{Heigh}}{{\text{t}}^{\text{2}}}({{\text{m}}^{\text{2}}}).$$

$${\text{GNRI}}\,=\,{\text{1}}.{\text{489 }} \times {\text{ Albumin }}\left( {{\text{g}}/{\text{L}}} \right)\,+\,{\text{41}}.{\text{7 }} \times {\text{ Current Weight }}\left( {{\text{kg}}} \right)/{\text{Ideal Weight }}\left( {{\text{kg}}} \right).$$

Ideal weight is calculated based on a BMI of 22 kg/m². If the current weight exceeds the ideal weight, the ratio is capped at 1. There were 4 grades of nutrition-related risk: major nutrition-related risk (GNRI: < 82), medium nutrition-related risk (GNRI: 82 to < 92), low nutrition-related risk (GNRI: 92 to ≤ 98), and no nutrition-related risk (GNRI: > 98)^16^. All the values used in GNRI calculation were obtained before surgery. The calculation method of ACCI is recorded in detail in Table supplementary 1.

### Statistical analysis

Data analysis was performed using SPSS 20.0, X-tile 3.6.1, and R 4.22 software. The study workflow is illustrated in the flow chart shown in Fig. 1B. Of the 389 enrolled patients, we randomly allocated them in a 2:1 ratio to a training group and a validation group using SPSS software. Baseline characteristics between the two groups were compared using SPSS, applying the chi-square test for categorical variables and the independent sample t-test for continuous variables. We began our study with a univariate Cox regression analysis using SPSS to identify potential prognostic factors among 21 variables. Significant factors were then included in a multivariate Cox regression to determine independent predictors of disease-free survival (DFS) and overall survival (OS). Using these independent factors, we developed two nomograms: one for predicting DFS and another for predicting OS. Finally, to validate the performance of the nomograms, additional statistical analyses were conducted using R software. This included calculating the receiver operating characteristic (ROC) curve, integrated discrimination improvement (IDI), net reclassification improvement (NRI), calibration curve, and decision curve analysis (DCA). A statistically significant difference was defined as *P* < 0.05.

### Risk stratification

Each variable included in the nomogram has a corresponding risk score. The risk scores for these variables were summed to obtain the overall risk score. For each case, there is an overall risk score in the prognostic model. The X-tile software was used to determine the best cut point of the total risk score, and the patients were divided into two subgroups of high and low risk to develop a prognostic stratification system. Kaplan-Meier method and log-rank test were used to compare DFS and OS in each risk subgroup.

## Results

### Clinical characteristics

In this study, 389 postoperative patients with PGC were analyzed, including 258 in the training cohort and 131 in the validation cohort. Table 1 presents an overview of the clinicopathological characteristics of the 389 patients involved in the study. A comparison of baseline characteristics between the training and validation cohorts indicated no statistically significant differences across all 21 variables (all *P* > 0.05).

The 3-year and 5-year DFS rates were 48.3% and 25.2%, respectively, while the 3-year and 5-year OS rates were 66.3% and 48.6%, respectively. The median age at diagnosis was 52 years (Interquartile Range (IQR): 39-68.5), with 41.1% of patients being female and 58.9% being male. The most prevalent pathology type was mucoepidermoid carcinoma (66.8%), followed by adenoid cystic carcinoma (15.2%) and follicular cell carcinoma (11.1%). In terms of tumor location, 47% of patients had tumors in the superficial lobe of the parotid gland, 39.1% had tumors in the deep lobe, and 13.9% had tumors affecting both lobes. A significant majority of patients (87.1%) were non-smokers. Most patients had an ECOG PS of 0–1 (77.6%), and 87.1% had negative surgical margins. VI was present in 8.5% of cases, while perineural invasion was observed in 18.5%. ENE was identified in 9.5% of patients. According to AJCC staging, 19.3% of patients were in stage I, 24.4% in stage II, 37% in stage III, and 19.3% in stages IVA and IVB. The median BMI was 21.6 kg/m², and the median hemoglobin and albumin levels were 102.9 g/L and 38.0 g/L, respectively. Regarding the GNRI, 38.8% of patients scored above 98, 22.1% scored between 92 and 98, 24.9% scored between 82 and 92, and 14.1% scored below 82. Additionally, 35.4% of patients received adjuvant radiotherapy.

### Nomogram risk models establishing

**Fig. 2:**
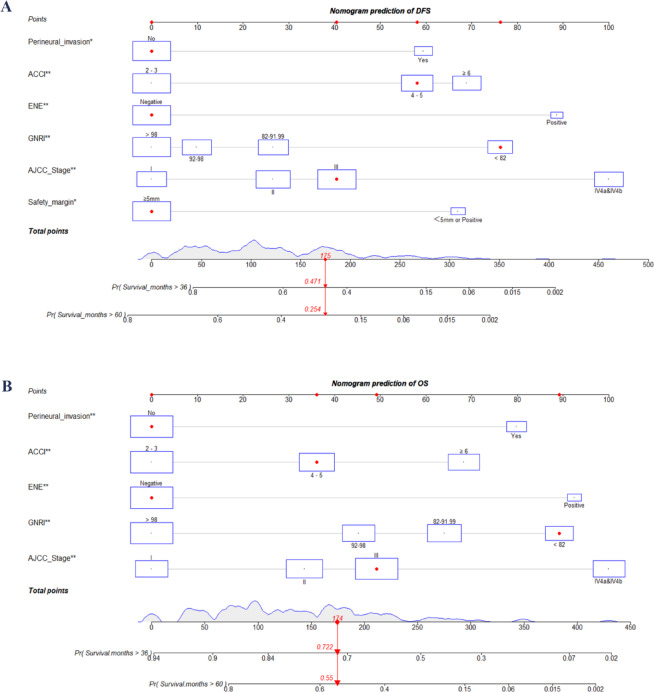
Nomogram risk models for predicting DFS (**A**) and OS (**B**) in postoperative PGC patients. ACCI, age-adjusted Charlson comorbidity index; AJCC, American Joint Committee on Cancer; DFS, disease-free survival; ECOG, eastern cooperative oncology group; ENE, extranodal extension; OS, overall survival, PS, performance status, PGC, parotid gland carcinoma.

Independent prognostic factors were identified using univariate and multivariate Cox regression analyses (Tables 2 and 3). These significant predictors were then incorporated into a nomogram to estimate DFS and OS. The variables included in the nomogram for predicting DFS were surgical margin, perineural invasion, ENE, ACCI, AJCC stage, and GNRI (see Fig. 2A; Table 2). Similarly, the predictors used to develop the nomogram for predicting OS included perineural invasion, ENE, ACCI, AJCC stage, and GNRI (see Fig. 2B; Table 3). Figure 2 also demonstrates how the prediction model can be utilized to estimate DFS and OS for individual patients.

### Validation

**Fig. 3:**
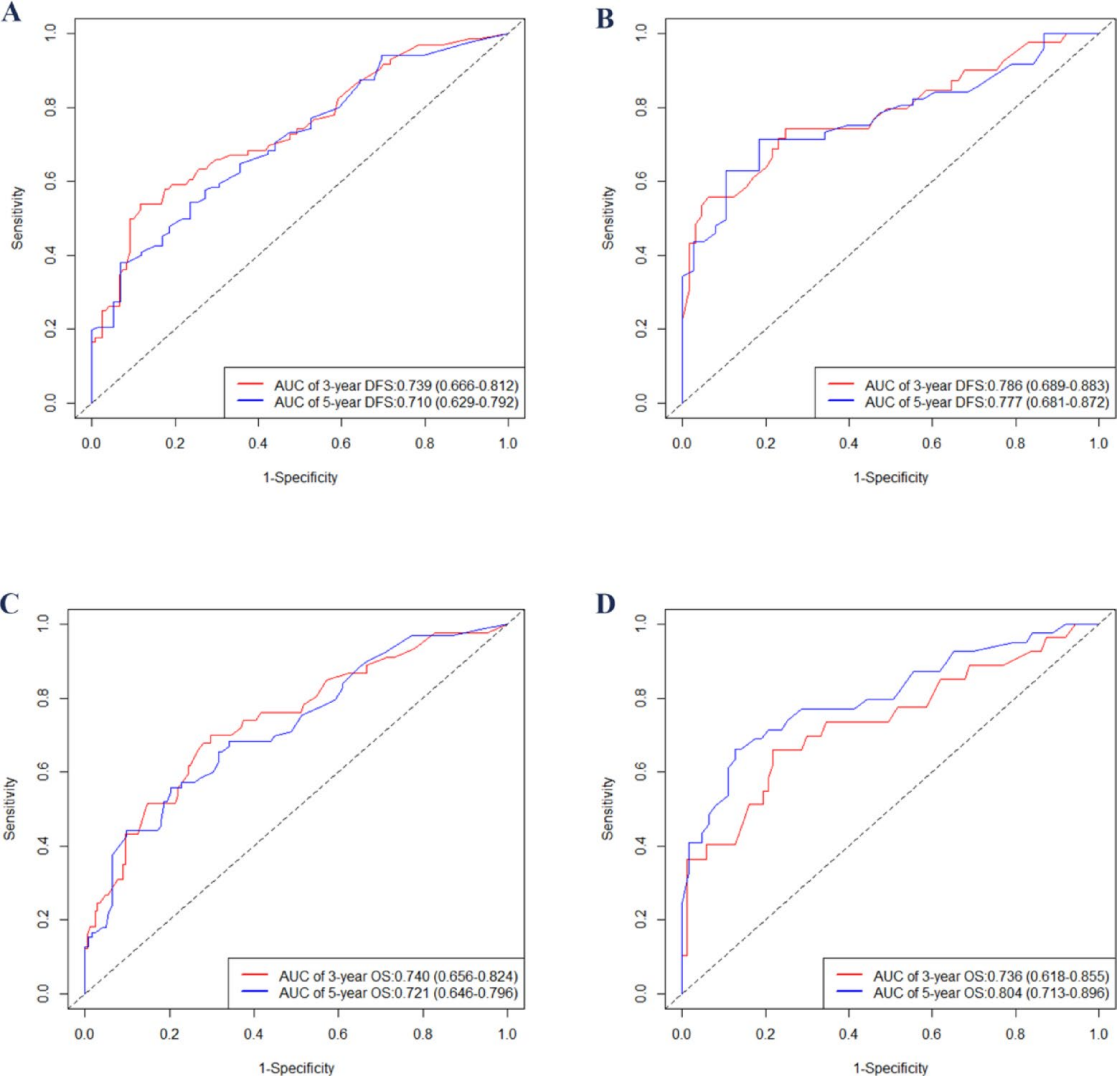
Time-dependent ROC curves for the two nomograms, showing AUC values for predicting 3- and 5-year DFS in the training set (A) and validation set (B), and 3- and 5-year OS in the training cohort (C) and validation cohort (D). AUC, area under curve; OS, overall survival; DFS, disease-free survival; ROC, receiver operating characteristic.

**Fig. 4:**
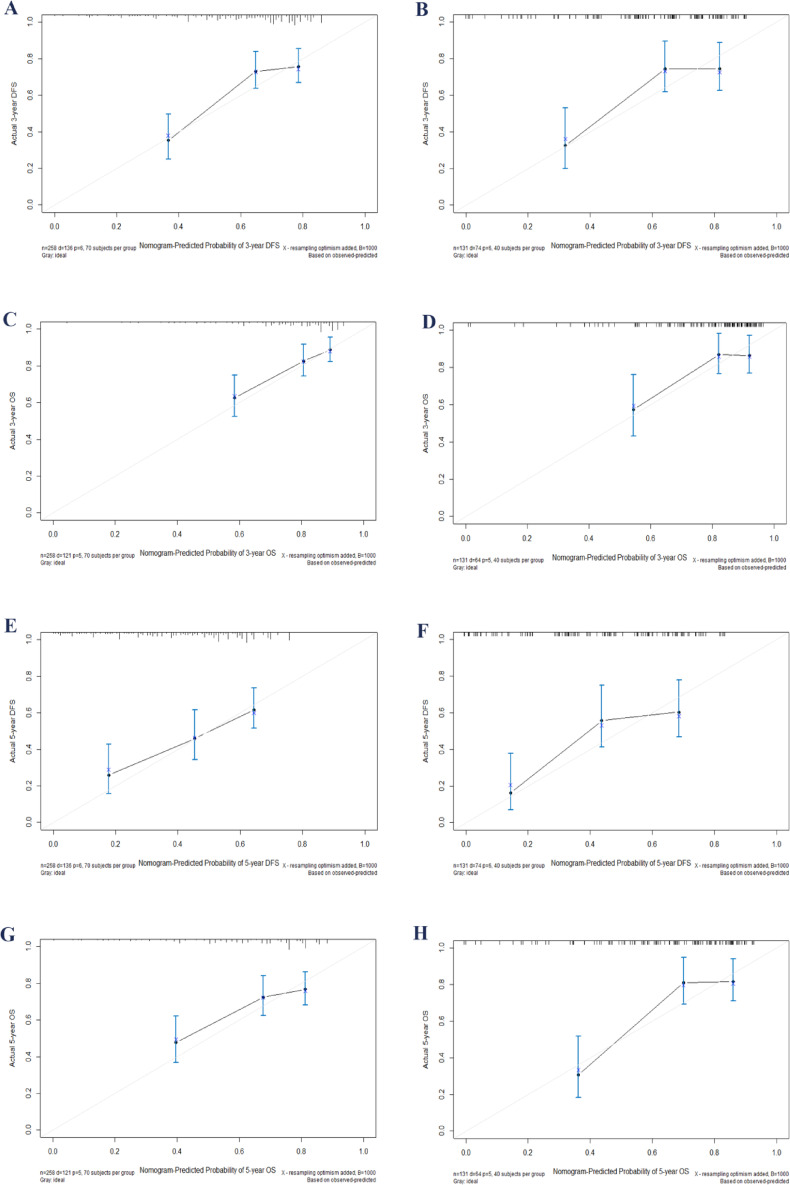
Calibration plots for 3- and 5-year survival in postoperative PGC patients: Plots **A** and **E** show 3- and 5-year DFS calibration, and plots **C** and **G** show OS calibration in the training set. Similarly, plots **B** and **F** show 3- and 5-year DFS, and plots **D** and **H** show OS calibration in the validation set. OS, overall survival; CI, confidence interval; DFS, disease-free survival; PGC, parotid gland carcinoma.

**Fig. 5:**
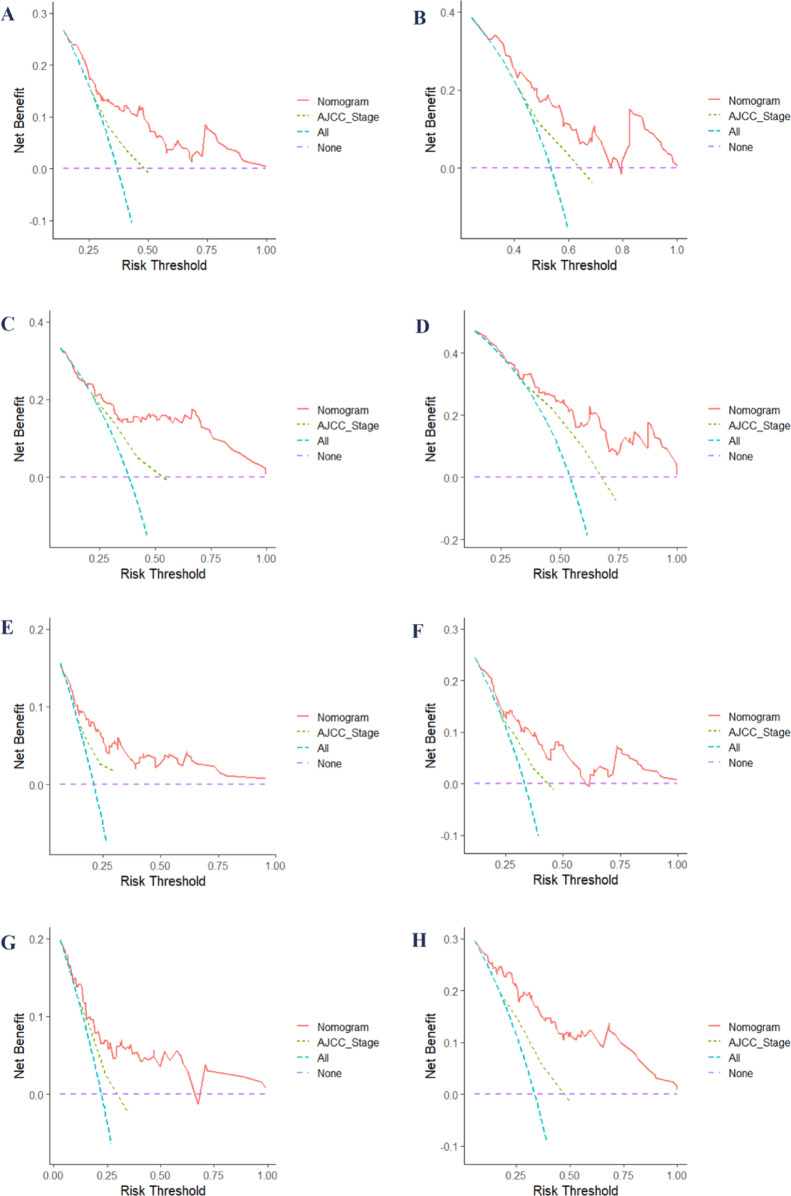
DCA for nomograms and AJCC staging in predicting DFS and OS: **A** and **B** show 3- and 5-year DFS DCA curves in the training cohort, while **C** and **D** display them for the validation cohort. Similarly, **E** and **F** show 3- and 5-year OS DCA curves in the training cohort, with **G** and **H** representing the validation cohort. AJCC, American Joint Committee on Cancer; DCA, decision curve analysis; DFS, disease-free survival; OS, overall survival.

After creating a nomogram for risk prediction, it is crucial to assess its accuracy and effectiveness using various indicators and visual tools. The area under the curve (AUC) values for the training set were impressive: 3-year disease-free survival (DFS) was 0.739, 5-year DFS was 0.710, 3-year overall survival (OS) was 0.740, and 5-year OS was 0.721, as illustrated in Fig. 3A and B. In the validation set, the AUC values remained high: 3-year DFS was 0.786, 5-year DFS was 0.777, 3-year OS was 0.736, and 5-year OS was 0.804, as shown in Fig. 3C and D. All AUCs exceeded 0.7, highlighting the model’s excellent discrimination ability. Calibration curves were closely aligned with the 45-degree diagonal, indicating a strong agreement between the predicted risk probabilities and the actual outcomes (Fig. 4). The concordance index (C-index) for the nomogram models in the training group was 0.712 for DFS and 0.697 for OS, while in the validation group, it was 0.730 for DFS and 0.722 for OS. Compared to the traditional AJCC staging system, the new model significantly improved the C-index (Table 4). Furthermore, all IDI and NRI values were greater than 0 in both the training and validation groups, demonstrating the enhanced discriminatory power and reclassification performance of the nomogram over AJCC staging. The results are summarized in Table 4. Additionally, the decision curve analysis (DCA) further supported the clinical utility of the new nomogram, indicating that it provides greater clinical benefit than AJCC staging across a range of thresholds (Fig. 5).

After performing 200 iterations of 10-fold cross-validation, the mean AUC increased to 0.780 at 3 years and 0.880 at 5 years For OS. The mean AUCs after 200 iterations of 10-fold cross-validation were 0.850 and 0.800 at 3 and 5 years, respectively. These findings suggest that the model demonstrates robust and consistent predictive ability, especially after cross-validation.

### Prognostic risk stratification

**Fig. 6:**
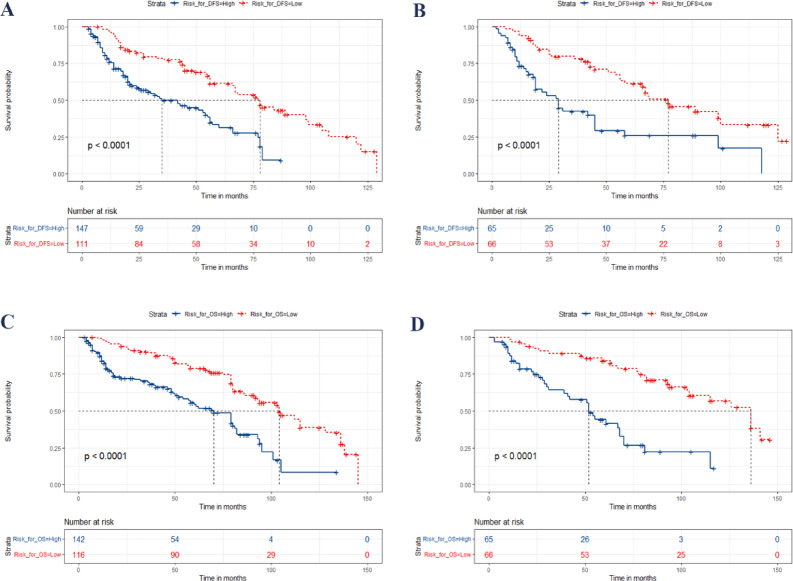
Kaplan-Meier Curves for Postoperative PGC Patients in Training and Validation Cohorts Based on the New Risk Stratification System. **A** and **B** depict the Kaplan-Meier curves for DFS in the training and validation cohorts, respectively, while **C** and **D** show the curves for OS. Blue and red curves correspond to the high-risk and low-risk groups, respectively. DFS, disease-free survival; OS, overall survival; OSCC, oral squamous cell cancer; PGC, parotid gland carcinoma.

The patients were categorized into two distinct subgroups, namely low-risk and high-risk, based on the prognostic total risk points and by utilizing X-tile 3.6.1 software to determine the optimal cut-off points. For DFS prediction, patients with risk points ≤ 103.2 were categorized as low-risk, while those with points > 103.2 were classified as high-risk. Similarly, for OS prediction, a score of ≤ 113.0 indicated low-risk, and > 113.0 indicated high-risk. The Kaplan-Meier survival curves (Fig. 6) demonstrated significant differences in both DFS and OS between these two subgroups, showing clear separation in survival outcomes for both the training and validation sets.

## Discussion

Malnutrition is estimated to account for up to 20% of cancer-related deaths, often eclipsing the impact of the tumor itself^22^. Patients with head and neck cancers are particularly vulnerable, with approximately two-thirds of this population experiencing malnutrition^23^. Among the numerous studies of head and neck tumors, there are few studies focusing on the nutritional status of PGC patients. Thus, evaluating the nutrition-related influencing factors of patients is a crucial aspect of the comprehensive management of PGC following surgery, as it can significantly impact survival outcomes^2^.

Currently, there is no standardized approach for assessing the nutritional status of cancer patients. Commonly used methods include the Mini Nutritional Assessment short-form, Nutritional Risk Screening 2002, Malnutrition Universal Screening Tool, Malnutrition Screening Tool, Short Nutritional Assessment Questionnaire, and various serum nutrition-related markers^24–26^. The nutritional status assessment tools above in clinical practice have several limitations. Firstly, these tools often rely on patient-reported feedback and active cooperation, combined with complex operations, making the assessment time-consuming and unsuitable for busy clinical environments. Furthermore, some tools are overly simplified, potentially underestimating the risk of malnutrition and failing to effectively screen high-risk patients. Finally, single serum nutritional markers are influenced by various factors, such as inflammation or acute illness, and cannot fully reflect the patient’s nutritional status. Therefore, it is crucial to perform a simple yet effective nutritional assessment before treatment to identify high-risk patients promptly. In contrast, the GNRI is calculated using basic parameters like weight, height, and serum albumin, making it easy to use while accurately reflecting a patient’s nutritional status^16^. Due to its simplicity and reliability, GNRI has become a valuable tool for quickly identifying patients at high risk of malnutrition, especially in time-sensitive clinical settings, offering clear advantages in efficiency.

The impact of GNRI on the prognosis of various tumors has become a prominent research topic in recent years. Rikako Kato et al.‘s study identified GNRI as an independent prognostic factor in metastatic colon cancer, with lower GNRI associated with significantly worse overall survival^27^. The results of another multivariate analysis involving 203 hepatocellular carcinoma patients showed that OS and recurrence-free survival were significantly worse in patients with low GNRI^28^. Pengjie Wu et al. conducted a study involving 458 postoperative upper tract urothelial carcinoma patients. Their retrospective analysis indicated that low GNRI was significantly associated with reduced survival and a higher incidence of postoperative complications compared to high GNRI^29^. A multicenter retrospective study in Japan involving 497 older patients with gastric cancer indicated that among various prognostic factors influencing OS and disease-specific survival, GNRI demonstrated the strongest predictive performance following curative gastrectomy^30^. In the study of prognosis of pancreatic cancer, a small-sample retrospective study on advanced pancreatic cancer also supports the conclusion that GNRI significantly impacts survival outcomes^31^. In prognostic studies of non-small cell lung cancer, a systematic review and meta-analysis indicate that GNRI is a key prognostic marker, with lower GNRI scores associated with an increased risk of reduced OS and DFS, making it a valuable tool for predicting survival^32^.

It is worth noting that GNRI has gained attention in the context of head and neck tumors, particularly for its role in predicting survival outcomes. The study by Yu Fujiwara et al. indicated that among older adults with locally advanced head and neck cancer undergoing concurrent chemoradiotherapy, a lower GNRI was significantly associated with worse OS, highlighting the importance of nutritional status as a prognostic factor, though no significant effect was observed on event-free survival^33^. Yu Ito et al. investigated the prognostic significance of preoperative GNRI in older adults (≥ 65 years) undergoing radical surgery for oral squamous cell carcinoma^34^, finding that lower GNRI values (≤ 93.7) were associated with significantly poorer OS and DFS outcomes, suggesting that GNRI assessment and nutritional interventions could enhance prognosis for high-risk patients. In terms of nasopharyngeal carcinoma research, Qing-Nan Tang et al. found that pretreatment GNRI is an independent prognostic factor in nasopharyngeal carcinoma patients, while the combination of GNRI and Epstein-Barr Virus DNA enabled effective categorization of patients into high-, medium-, and low-risk groups^35^. However, no studies have specifically investigated the impact of the GNRI on the prognosis of PGC to date. Therefore, our research serves as an important addition to existing literature. In our study, patients with low GNRI exhibited poorer OS and DFS. More importantly, our multivariate Cox regression analysis revealed that GNRI is an independent prognostic factor affecting postoperative outcomes in PGC patients. Additionally, our study developed two nomograms based on GNRI and other clinicopathological variables to predict DFS and OS. This is the first GNRI-based prognostic model specifically designed to predict the prognosis of PGC patients after surgery, making it highly novel. Compared to the traditional 8th edition AJCC staging system, our new models showed a significant improvement in the C-index, indicating a marked enhancement in predictive accuracy. This underscores the model’s ability to better predict outcomes in PGC patients, highlighting the significant prognostic value of GNRI and providing a more effective tool for guiding clinical decisions in postoperative care.

Apart from GNRI and AJCC staging, additional variables included in the nomogram following multivariate Cox regression analysis were surgical margin, perineural invasion, ENE, and ACCI. The influence of these predictors on the prognosis of PGC patients has been well-documented in previous literature, and our findings are largely consistent with those studies^3,36–41^. Therefore, incorporating these variables into the nomogram is well-supported by existing references, enhancing the reliability and predictive power of the model for PGC prognosis.

While this study provides valuable insights, it also has several significant limitations. First, the retrospective nature of the analysis may introduce selection bias, limiting the applicability of the findings to broader populations. Secondly, as the research was conducted at only two regional medical centers, the results may not reflect the diverse clinical practices present in different settings. Thirdly, this study did not compare the predictive capabilities of GNRI with other nutritional assessment tools. Fourthly, although preoperative nutritional status was considered, other potential confounding variables, such as postoperative complications, were not taken into account, which may affect the outcomes. Finally, while cross-validation is commonly used in model evaluation, considering the sample size and data characteristics of this study, we implemented an independent partitioning of the training and validation sets combined with a rigorous model validation process to effectively mitigate the risks of data leakage and overfitting. In this study, the training and validation sets were segmented using a systematic approach, and the model’s performance was independently validated using metrics such as the C-index and AUC. We believe that this methodology ensures the robustness and reliability of the model, as evidenced by its effectiveness in real-world clinical applications. Future studies should adopt multicenter designs and consider additional confounding factors to enhance generalizability and robustness.

## Conclusion

GNRI is a valuable prognostic factor for postoperative PGC patients. The GNRI-based nomogram developed in this study demonstrates superior predictive accuracy for both DFS and OS compared to traditional AJCC staging, offering significant clinical potential.

**Table 1:** Clinical information of postoperative PGC patients in the training and validation groups.

Characteristics	All Patients (*n* = 389) *N* (%)	Training cohort (*n* = 258) *N* (%)	Validation cohort (*n* = 131) *N* (%)	*P*
Age at diagnosis (years)				0.131
Median (IQR)	52 (39-68.5)	51 (39.8–67)	52 (38–69)	
Gender				0.267
Female	160 (41.1%)	101 (39.1%)	59 (45.0%)	
Male	229 (58.9%)	157 (60.9%)	72 (55.0%)	
Pathology				0.793
Mucoepidermoid carcinoma	260 (66.8%)	170 (65.9%)	90 (68.7%)	
Adenoid cystic carcinoma	59 (15.2%)	42 (16.3%)	17 (13.0%)	
Follicular cell carcinoma	43 (11.1%)	29 (11.2%)	14 (10.7%)	
Others^a^	27 (6.9%)	17 (6.6%)	10 (7.6%)	
Grade				0.107
I	125 (32.1%)	92 (35.7%)	33 (25.2%)	
II	130 (33.4%)	83 (32.2%)	47 (35.9%)	
III	134 (34.4%)	83 (32.2%)	51 (38.9%)	
Tumor location				0.213
Superficial	183 (47.0%)	128 (49.6%)	55 (42.0%)	
Deep	152 (39.1%)	99 (38.4%)	53 (40.5%)	
Superficial & Deep	54 (13.9%)	31 (12.0%)	23 (17.6%)	
Smoking				0.488
No	339 (87.1%)	227 (88.0%)	112 (85.5%)	
Yes	50 (12.9%)	31 (12.0%)	19 (14.5%)	
ECOG PS score				0.936
0–1	302 (77.6%)	200 (77.5%)	102 (77.9%)	
2	87 (22.4%)	58 (22.2%)	29 (22.1%)	
Surgical margin				0.710
Negative	339 (87.1%)	226 (87.6%)	113 (86.3%)	
Positive	50 (12.9%)	32 (12.4%)	18 (13.7%)	
VI				0.135
No	356 (91.5%)	240 (93.0%)	116 (88.5%)	
Yes	38 (8.5%)	18 (7.0%)	15 (11.5%)	
Perineural invasion				0.835
No	317 (81.5%)	211 (81.8%)	106 (80.9%)	
Yes	72 (18.5%)	47 (18.2%)	25 (19.1%)	
ENE				0.866
Negative	352 (90.5%)	233 (90.3%)	119 (90.8%)	
Positive	37 (9.5%)	25 (9.7%)	12 (9.2%)	
AJCC Stage				
I	75 (19.3%)	52 (20.2%)	23 (17.6%)	0.105
II	95 (24.4%)	70 (27.1%)	25 (19.1%)	
III	144 (37.0%)	85 (32.9%)	59 (45.0%)	
IVA & IVB	75 (19.3%)	51 (19.8%)	24 (18.3%)	
BMI (kg/m^2^)				0.274
Median (IQR)	21.6 (19.3–25.0)	21.0 (19.1–24.7)	22.0 (19.6–25.6)	
Hemoglobin (g/L)				0.304
Median (IQR)	102.9 (92.5-118.3)	102.7 (92.1–118.0)	103.0 (92.7-118.6)	
Albumin (g/L)				0.131
Median (IQR)	38.0 (34.7–48.1)	36.9 (33.0–48.0)	39.0 (34.1–48.0)	
GNRI				0.342
> 98	151 (38.8%)	97 (37.6%)	54 (41.2%)	
92 to ≤ 98	86 (22.1%)	58 (22.5%)	28 (21.4%)	
82 to < 92	97 (24.9%)	61 (23.6%)	36 (27.5%)	
< 82	55 (14.1%)	42 (16.3%)	13 (9.9%)	
ACCI				0.620
2–3	167 (42.9%)	115 (44.6%)	52 (39.7%)	
4–5	126 (32.4%)	80 (31.0%)	46 (35.1%)	
≥ 6	96 (24.7%)	63 (24.4%)	33 (25.2%)	
Type of resection				0.423
Total parotidectomy	200 (54.7%)	136(56.4%)	64 (50.5%)	
Radical parotidectomy	164 (40.1%)	107 (38.6%)	57(43.7%)	
Superficial parotidectomy	25 (5.2%)	15 (5.0%)	10 (5.8%)	
Adjuvant radiotherapy				0.348
No	250 (64.3%)	170 (65.9%)	80 (61.1%)	
Yes	139 (35.4%)	88 (34.1%)	51 (38.9%)	
DFS (months)				0.119
Median (IQR)	32 (15–61)	30.5 (14–60)	34 (16–63)	
OS (months)				0.272
Median (IQR)	58 (21-84.5)	58 (20-82.3)	59 (22–86)	

Others^a^, squamous carcinoma, ductal carcinoma of the salivary gland, papillary cystic carcinoma.

ACCI, age-adjusted Charlson comorbidity index; AJCC, American Joint Committee on Cancer; BMI, body mass index; DFS, disease-free survival; ECOG, eastern cooperative oncology group; ENE, extranodal extension; GNRI, Geriatric Nutritional Risk Index,; IQR, interquartile range; OS, overall survival; PGC, parotid gland carcinoma; PS, performance status; VI, vascular invasion.

**Table 2:** Univariate and multivariate analyses of clinicopathologic parameters in postoperative patients with PGC for predicting DFS in the training group.

Characteristics	Univariate analysis		Multivariate analysis	
	HR (95% CI)	*P*	HR (95% CI)	*P*
Age at diagnosis (years)	1.013 (1.003–1.023)	**0.014**	0.999 (0.986–1.012)	0.878
Gender				
Female	Reference			
Male	0.947 (0.672–1.334)	0.755		
Pathology				
Mucoepidermoid carcinoma	Reference			
Adenoid cystic carcinoma	1.311 (0.993–1.697)	0.446		
Follicular cell carcinoma	0.753 (0.693–1.197)	0.160		
Others^a^	1.112 (0.956–1.497)	0.357		
Grade				
I	Reference		Reference	
II	1.357 (0.885–2.081)	0.162	1.070 (0.674–1.699)	0.773
III	1.532 (1.021–2.298)	**0.039**	1.382 (0.885–2.158)	0.154
Tumor location				
Superficial	Reference			
Deep	1.341 (0.926–1.942)	0.170		
Superficial & Deep	1.630 (0.950–2.798)	0.076		
Smoking				
No	Reference			
Yes	1.493 (0.917–2.431)	0.107		
ECOG PS score				
0–1	Reference		Reference	
2	1.429 (0.971–2.104)	**0.042**	1.424 (0.936–2.168)	0.099
Surgical margin				
Negative	Reference		Reference	
Positive	2.169 (1.339–3.541)	**0.002**	1.760 (1.017–3.047)	**0.044**
VI				
No	Reference		Reference	
Yes	1.791 (1.045–3.071)	**0.034**	1.083 (0.556–2.109)	0.816
Perineural invasion				
No	Reference		Reference	
Yes	1.973 (1.313–2.966)	**0.001**	1.696 (1.055–2.725)	**0.029**
ENE				
Negative	Reference		Reference	
Positive	2.205 (1.262–3.851)	**0.005**	2.277 (1.246–4.163)	**0.007**
AJCC Stage				
I	Reference		Reference	
II	1.044 (0.622–1.755)	0.870	1.190 (0.684–1.850)	0.526
III	1.858 (1.150–3.002)	**0.011**	1.429 (0.856–2.386)	0.173
IVA & IVB	2.114 (1.225–3.646)	**0.007**	2.339 (1.321–4.141)	**0.004**
BMI (kg/m^2^)	0.989 (0.893–1.897)	0.640		
Hemoglobin (g/L)	0.998 (0.990–1.007)	0.710		
Albumin (g/L)	1.001 (0.982–1.021)	0.892		
GNRI				
> 98	Reference			
92–98	1.074 (0.665–1.734)	0.771	1.190 (0.695–2.036)	0.643
82-91.99	1.506 (0.958–2.366)	0.076	1.429 (0.856–2.386)	0.157
< 82	2.103 (1.275–3.467)	**0.004**	2.030 (1.212-3.400)	**0.007**
ACCI				
2–3	Reference			
4–5	1.574 (1.064–2.330)	**0.023**	1.656 (1.102–2.487)	**0.015**
≥ 6	1.850 (1.176–2.910)	**0.008**	1.827 (1.144–2.918)	**0.012**
Type of resection				
Total parotidectomy	Reference			
Radical parotidectomy	0.987 (0.875–1.331)	0.128		
Superficial parotidectomy	1.250 (1.016–1.610)	0.254		
Adjuvant radiotherapy				
No	Reference			
Yes	0.815 (0.571–1.163)	0.259		

Others^a^, squamous carcinoma, ductal carcinoma of the salivary gland, papillary cystic carcinoma.

ACCI, age-adjusted Charlson comorbidity index; AJCC, American Joint Committee on Cancer; BMI, body mass index; DFS, disease-free survival; ECOG, eastern cooperative oncology group; ENE, extranodal extension; GNRI, Geriatric Nutritional Risk Index,; IQR, interquartile range; PGC, parotid gland carcinoma; PS, performance status; VI, vascular invasion.

**Table 3:** Univariate and multivariate analyses of clinicopathologic parameters in postoperative patients with PGC for predicting OS in the training group.

Characteristics	Univariate analysis		Multivariate analysis	
	HR (95% CI)	*P*	HR (95% CI)	*P*
Age at diagnosis (years)	1.014 (1.004–1.025)	**0.007**	1.003 (0.990–1.016)	0.659
Gender				
Female	Reference			
Male	0.905 (0.630–1.301)	0.591		
Pathology				
Mucoepidermoid carcinoma	Reference			
Adenoid cystic carcinoma	1.218 (0.989–1.611)	0.540		
Follicular cell carcinoma	0.803 (0.712–1.208)	0.235		
Others^a^	1.188 (0.934–1.403)	0.367		
Grade				
I	Reference			
II	1.220 (0.776–1.919)	0.389		
III	1.532 (1.001–2.347)	0.050		
Tumor location				
Superficial	Reference			
Deep	1.384 (0.935–2.047)	0.104		
Superficial & Deep	1.415 (0.771–2.597)	0.236		
Smoking				
No	Reference		Reference	
Yes	1.733 (1.059–2.834)	**0.029**	1.596 (0.962–2.649)	0.245
ECOG PS score				
0–1	Reference			
2	1.277 (0.844–1.933)	0.248		
Surgical margin				
Negative	Reference		Reference	
Positive	2.037 (1.339–3.541)	**0.006**	1.557 (0.871–2.783)	0.135
VI				
No	Reference			
Yes	1.641 (0.921–2.922)	0.093		
Perineural invasion				
No	Reference		Reference	
Yes	2.034 (1.330–3.110)	**0.001**	2.286 (1.453–2.598)	**< 0.001**
ENE				
Negative	Reference		Reference	
Positive	2.636 (1.474–4.712)	**0.001**	2.487 (1.327–4.662)	**0.004**
AJCC Stage				
I	Reference		Reference	
II	1.062 (0.618–1.827)	0.827	1.201 (0.690–2.090)	0.517
III	1.645 (1.002-2.700)	0.049	1.293 (0.769–2.175)	0.333
IVA & IVB	1.895 (1.066–3.371)	**0.039**	2.239 (1.231–4.017)	**0.008**
BMI (kg/m^2^)	0.964 (0.918–1.012)	0.137		
Hemoglobin (g/L)	0.991 (0.982–1.001)	0.068		
Albumin (g/L)	0.983 (0.962–1.003)	0.100		
GNRI				
> 98	Reference		Reference	
92 to ≤ 98	1.529 (0.931–2.511)	0.093	1.539 (0.917–2.583)	0.103
82 to < 92	1.909 (1.170–3.116)	**0.010**	1.615 (0.966-2.700)	0.067
< 82	2.346 (1.387–3.970)	**0.001**	2.253 (1.372–4.034)	**0.002**
ACCI				
2–3	Reference		Reference	
4–5	1.306 (0.857–1.990)	0.214	1.452 (0.938–2.247)	0.094
≥ 6	2.035 (1.247–3.250)	**0.003**	1.957 (1.205–3.179)	**0.007**
Type of resection				
Total parotidectomy	Reference			
Radical parotidectomy	1.012 (0.895–1.234)	0.188		
Superficial parotidectomy	1.351 (1.134–1.766)	0.424		
Adjuvant radiotherapy				
No	Reference			
Yes	0.735 (0.503–1.075)	0.112		

Others^a^, squamous carcinoma, ductal carcinoma of the salivary gland, papillary cystic carcinoma.

ACCI, age-adjusted Charlson comorbidity index; AJCC, American Joint Committee on Cancer; BMI, body mass index; ECOG, eastern cooperative oncology group; ENE, extranodal extension; GNRI, Geriatric Nutritional Risk Index; IQR, interquartile range; OS, overall survival; PGC, parotid gland carcinoma; PS, performance status; VI, vascular invasion.

**Table 4:** The IDI, NRI, and C-index of the nomograms and AJCC stage in OS and DFS prediction for PGC patients after surgery.

Index	Training cohort		*P*	Validation cohort		*P*
	Estimate	95%CI		Estimate	95%CI	
NRI (vs. AJCC Stage system)						
For 3-year OS	0.317	0.124–0.445	**< 0.001**	0.321	0.053–0.504	**< 0.001**
For 5-year OS	0.310	0.128–0.444	**< 0.001**	0.380	0.162–0.582	**< 0.001**
For 3-year DFS	0.347	0.173–0.496	**< 0.001**	0.398	0.202–0.560	**< 0.001**
For 5-year DFS	0.302	0.107–0.447	**< 0.001**	0.302	0.109–0.528	**< 0.001**
IDI (vs. AJCC Stage system)						
For 3-year OS	0.107	0.053–0.192	**< 0.001**	0.165	0.050–0.336	**< 0.001**
For 5-year OS	0.121	0.055–0.208	**< 0.001**	0.201	0.096–0.331	**< 0.001**
For 3-year DFS	0.110	0.052–0.204	**< 0.001**	0.161	0.067–0.277	**< 0.001**
For 5-year DFS	0.090	0.033–0.174	**< 0.001**	0.141	0.034–0.270	**< 0.001**
C-index						
The nomogram (DFS)	0.712	0.661–0.763		0.730	0.659–0.801	
The nomogram (OS)	0.697	0.650–0.744		0.722	0.657–0.782	
The AJCC Stage (DFS)	0.621	0.562–0.680		0.641	0.572–0.710	
The AJCC Stage (OS)	0.621	0.566–0.675		0.638	0.575–0.701	

AJCC, American joint committee on cancer; CI, confidence interval; C-index, concordance index; DFS, disease-free survival; IDI, integrated discrimination improvement; NRI, net reclassification index; OS, overall survival; PGC, parotid gland cancer.

## Electronic supplementary material

Below is the link to the electronic supplementary material.

Supplementary Material 1

## Data Availability

The data in this article cannot be publicly shared due to concerns regarding patient privacy and data security. However, interested parties may request access to the data from the corresponding author upon reasonable request.

## Abbreviations

ACCI: Age-Adjusted Charlson Comorbidity Index
AJCC: American Joint Committee on Cancer
AUC: Area under curve
BMI: Body mass index
C-index: Concordance index
CI: Confidence interval
CRT: Conformal radiotherapy
DCA: Decision curve analysis
DFS: Disease-free survival
ECOG: Eastern cooperative oncology group
ENE: Extranodal extension
GNRI: Geriatric Nutritional Risk Index
IDI: Integrated discrimination improvement
IMRT: Intensity-modulated radiotherapy
IQR: Interquartile range
NRI: Net reclassification improvement
OS: Overall survival
PS: Performance status
PGC: Parotid gland carcinoma
ROC: Receiver operating characteristic
VMAT: Volumetric-modulated arc therapy
VI: Vascular invasion
