# Supplementary material for: The prognostic significance of the geriatric nutritional risk index in postoperative parotid gland carcinoma
Source: Sci Rep. 2025 Jul 1;15:22309. doi: 10.1038/s41598-025-08609-8 (PMC12217681; doi:10.1038/s41598-025-08609-8)
Supplement: Supplementary file 1 — Supplementary Material 1 [file 41598_2025_8609_MOESM1_ESM.docx]

| **Table Supplementary 1**. Calculation method of the Age-adjusted Charlson Comorbidity Index. | |
| --- | --- |
| **Clinical conditions included in the score** | **Scores** |
|  | **For Disease** |
| Cerebrovascular disease; Chronic pulmonary disease; Congestive heart failure; Connective tissue disease; Dementia; Diabetes; Mild liver disease; Myocardial infarction; Peripheral vascular disease; Ulcer disease | 1 points |
| Any tumor; Diabetes with endo organ damage; Hemiplegia; Leukemia; Lymphoma; Moderate or severe renal disease | 2 points |
| Moderate or severe liver disease | 3 points |
| Acquired immune deficiency syndrome; Metastatic solid tumor | 6 points |
|  | **For Age** |
| Each decade over age 40 years (up to 4 points) | 1 points |
